# Change of Detrusor Contractility in Patients with and without Bladder Outlet Obstruction at Ten or More Years of follow-up

**DOI:** 10.1038/s41598-019-55386-2

**Published:** 2019-12-11

**Authors:** Sheng-Fu Chen, Cheng-Ling Lee, Hann-Chorng Kuo

**Affiliations:** Department of Urology, Buddhist Tzu Chi General Hospital and Tzu Chi University, Hualien, Taiwan

**Keywords:** Bladder, Urological manifestations

## Abstract

To analyze the change of detrusor contractility by investigating urodynamic characteristics with long term follow-up. This study retrospectively reviewed 166 lower urinary tract symptoms patients without bladder outlet obstruction (BOO) and 63 patients with BOO who underwent repeated urodynamic studies at the first time and more than 10 years later. The urodynamic parameters, bladder contractility index (BCI), and BOO index (BOOI) were compared before and after. As time goes by, detrusor pressure at maximum flow rate (PdetQmax) significantly decreased and post-void residual (PVR) volume significantly increased in both men and women. Full sensation, urge sensation, voided volume, and BCI significantly decreased. We also compared men with and without BOO, PdetQmax, maximum flow rate (Qmax), voided volume, and BCI all significantly decreased in both groups without difference. PVR increased greater in men with BOO after >10 years significantly (p = 0.036). Women with detrusor overactivity (DO) under antimuscarinic showed no significant BCI change compared to patients without DO (p = 0.228). Detrusor contractility decreases in men and women after >10 years of follow-up. However, this finding suggests that patients with BOO or DO under adequate medical treatment, detrusor contractility is not aggravated over 10 or more years of follow-up.

## Introduction

Lower urinary tract symptoms (LUTS) show age-related alterations in both men and women without any underlying disease leading to micturition disorder^1^. Most studies of LUTS in the community or hospital-based populations indicate that an increase in LUTS with age is not sex specific^2^. Several studies have shown bladder contractility decreased by age and detrusor contractility significantly deteriorated in older women^3,4^. Zimmern *et al*. found bladder hypocontractility was more likely in women 65 years old or older in 2 large cohorts of women planning for stress urinary incontinence surgery^5^. However, longitudinal long-term follow-up studies focus on this issue are rare.

On the other hand, elderly men are usually equivocal due to benign prostate hyperplasia (BPH), which usually causes bladder outlet obstruction (BOO). This obstruction may lead to higher voiding pressure to overcome the increased urethral resistance which seems that increasing bladder contractility by age. Previously, investigators have reported that aging does not impair detrusor contractility in men^6^. Thomas *et al*. found the detrusor contractility in men with BOO was not significantly affected urodynamically in the long term follow-up^7^.

Beltrame supposed that older men with LUTS had significant decreases in voiding efficiency, which might be due to storage dysfunction rather than impaired detrusor contractility or bladder outlet obstruction^8^. Due to lack of report of long term follow-up of the change of detrusor contractility, we tried to investigate a cohort of men and women who had undergone videourodynamic studies (VUDS) before and after more than 10 years to evaluate the changes in detrusor contractility over time.

## Results

In this study, 166 patients were enrolled, including 49 men (mean age, 63.8 ± 13.2 years) and 117 women (mean age, 53.2 ± 13.1 years) without BOO. The mean follow-up period was 13.21 years (range, 10–20 years). Table 1 showed the urodynamic parameter change in man and women. After over 10 years follow-up, detrusor pressure at maximum flow rate (PdetQmax) decreased significantly and post-void residual (PVR) increased significantly in both men and women over time. First sensation (FS), urge sensation (US), and voided volume significantly decreased during follow-up. Bladder contractility index (BCI) also decreased significantly in men and women, but no significant difference was noted between men and women patients. In these non-BOO patients, bladder outlet obstruction index (BOOI) did not showed significant difference after follow-up in both sexes. Although BOOI was used in men, the value in women could also be referred to voiding pressure and uroflow relevance. We also compared the change in BCI by age (≥65 versus <65 years old), and both sexes showed no significant difference. (P = 0.065 in man and P = 0.472 in women).

**Table 1:** Changes of urodynamic parameters between baseline and over 10 years later in patients without bladder outlet obstruction.

Characteristic	Time	Male (n = 49)	Female (n = 117)	P value
FSF (mL)	Baseline	120.59 ± 57.31	133.56 ± 61.98	0.982
	Follow-up	120.76 ± 65.15	134.01 ± 67.54	
FS (mL)	Baseline	214.31 ± 97.33	230.53 ± 87.87	0.427
	Follow-up	175.82 ± 88.53*	207.26 ± 84.69*	
US (mL)	Baseline	296.69 ± 108.95	301.3 ± 103.62	0.104
	Follow-up	205.35 ± 103.69*	246.3 ± 107.09*	
CBC (mL)	Baseline	327.14 ± 131.02	341.7 ± 152.4	0.224
	Follow-up	257.43 ± 128.05*	309.7 ± 141.0	
Compliance	Baseline	64.32 ± 76.59	79.61 ± 83.03	0.965
	Follow-up	61.81 ± 71.68	76.3 ± 79.61	
PdetQmax (cmH_2_O)	Baseline	42.35 ± 26.51	23.11 ± 14.11	0.07
	Follow-up	30.57 ± 15.07*	17.88 ± 13.18*	
Qmax (mL/s)	Baseline	12.27 ± 5.52	15.26 ± 9.19	0.256
	Follow-up	8.43 ± 4.09*	13.34 ± 10.34	
Volume (mL)	Baseline	295.41 ± 140.26	305.1 ± 139.33	0.265
	Follow-up	203.33 ± 123.26*	243.2 ± 142.71*	
PVR mL)	Baseline	31.73 ± 42.52	36.58 ± 78.1	0.644
	Follow-up	54.10 ± 76.47*	66.33 ± 106.89*	
BCI	Baseline	103.67 ± 42.29	99.49 ± 46.39	0.041
	Follow-up	70.67 ± 26.7*	81.17 ± 37.73*	
BOOI	Baseline	18.22 ± 27.28	−6.14 ± 24.78	0.403
	Follow-up	14.37 ± 18.14	−6.31 ± 22.6	

*p-value < 0.05 comparison of baseline and >10 years.

BOO: bladder outlet obstruction FSF: first sensation of filling, FS: full sensation, US: urge sensation, Pdet: detrusor pressure, Qmax: maximum flow rate, PVR: post-void residual, CBC: cystometric bladder capacity, BCI: bladder contractility index, BOOI: BOO index.

Another 54 men (mean age, 67.0 ± 9.5 years) with proven bladder outlet obstruction (BOO) who had undergone first videourodynamic study (VUDS) and follow-up of 10 or more years (mean follow-up duration, 13.2 ± 2.6 years) were also enrolled in the final statistical analysis. All of them were treated with long-term alpha-blocker therapy with or without a 5-alpha-reductase inhibitor. They underwent repeat VUDS because of persistent LUTS. We compared the men with BOO vs non- BOO and found a significant decrease in PdetQmax, Qmax, voided volume, PVR, and BCI in both groups. However, no significant difference was found between the groups. Only PVR significantly increased in men with BOO compared to men without BOO after 10 or more years of follow-up (p = 0.036) (Table 2).

**Table 2:** Changes of urodynamic parameters between men with and without bladder outlet obstruction over 10 years follow-up.

Characteristic	Time	Men BOO (n = 54)	Men Non-BOO (n = 49)	P value
PdetQmax (cmH_2_O)	Baseline	55.6 ± 27.4	42.4 ± 26.5	0.867
	Follow-up	42.9 ± 27.3*	30.6 ± 15.1*	
Qmax (mL/s)	Baseline	10.1 ± 4.07	12.3 ± 5.52	0.585
	Follow-up	6.87 ± 4.31*	8.43 ± 4.09*	
Volume (mL)	Baseline	264.4 ± 129.6	295.4 ± 140.3	0.619
	Follow-up	157.3 ± 102.3*	203.3 ± 123.3*	
PVR (mL)	Baseline	41.1 ± 56.02	31.7 ± 6.07	0.036**
	Follow-up	103.3 ± 130.1*	54.1 ± 10.9*	
BCI	Baseline	105.9 ± 32.5	103.7 ± 6.04	0.577
	Follow-up	77.3 ± 35.2*	70.7 ± 3.81*	
BOOI	Baseline	35.3 ± 29.5	18.2 ± 3.90	0.714
	Follow-up	29.2 ± 28.4	14.37 ± 2.59	

*p-value < 0.05 comparison of baseline and >10 years.

**p-value < 0.05 comparison of BOO and non-BOO groups.

BOO: bladder outlet obstruction, Pdet: detrusor pressure, Qmax: maximum flow rate, PVR: post-void residual, BCI: bladder contractility index, BOOI: BOO index.

Finally, we compared the women with DO vs non-DO at baseline in Table 3. Of them, 43 women (mean age, 56.4 ± 14.0 years) with DO and 74 women (mean age, 51.3 ± 12.2 years) without DO were included in the analysis. Patients with DO at baseline were treated with antimuscarinic agents. The urodynamic studies revealed FSF, FS, US, CBC, and voided volume all decreased significantly in women with DO during follow-up. In addition, voided volume, PVR, and BCI decreased significantly in both groups. The change in detrusor contractility defined by the change in BCI did not differ between the groups (p = 0.228).

**Table 3:** Changes of urodynamic parameters between women with and without detrusor overactivity over 10 years later.

Characteristic	Time	DO (n = 43)	Non DO (n = 74)	P value
FSF (mL)	Baseline	133.84 ± 53.10	133.39 ± 66.94	0.031**
	Follow-up	114.70 ± 50.84	145.23 ± 73.58	
FS (mL)	Baseline	228.49 ± 84.06	231.72 ± 90.55	0.006**
	Follow-up	169.23 ± 67.85*	229.36 ± 86.03	
US (mL)	Baseline	298.21 ± 106.75	303.12 ± 102.4	0.003**
	Follow-up	196.05 ± 89.68*	275.49 ± 106.0	
CBC (mL)	Baseline	319.86 ± 117.34	354.45 ± 168.8	0.05**
	Follow-up	244.30 ± 95.14*	347.64 ± 149.6	
Compliance	Baseline	71.11 ± 58.35	84.55 ± 94.50	0.81
	Follow-up	64.69 ± 79.21	83.05 ± 79.59	
PdetQmax (cmH_2_O)	Baseline	26.09 ± 17.84	21.38 ± 11.17	0.706
	Follow-up	21.65 ± 14.75	15.69 ± 11.73*	
Qmax (mL/s)	Baseline	16.14 ± 7.04	14.74 ± 10.25	0.115
	Follow-up	12.12 ± 5.83*	14.05 ± 12.21	
Volume (mL)	Baseline	300.56 ± 117.67	307.69 ± 151.2	0.103
	Follow-up	207.67 ± 96.06*	263.7 ± 160.8*	
PVR (mL)	Baseline	19.07 ± 23.28	46.76 ± 95.37	0.246
	Follow-up	36.63 ± 48.14*	83.59 ± 126.4*	
BCI	Baseline	106.81 ± 33.75	95.23 ± 52.10	0.228
	Follow-up	82.23 ± 25.62*	80.55 ± 43.40*	

*p-value < 0.05 comparison of baseline and >10 years.

**p-value < 0.05 comparison of BOO and non-BOO group.

DO: detrusor overactivity; FSF: first sensation of filling, FS: full sensation, US: urge sensation, Pdet: detrusor pressure, Qmax: maximum flow rate, PVR: post-void residual, CBC: cystometric bladder capacity, BCI: bladder contractility index, BOOI: BOO index.

## Discussion

In this longitudinal study, the most important finding is that BCI decreased significantly with age in both sexes. Voided volume decreased significantly, and PVR increased significantly with age. Additionally, when we focused on BCI, which indicates detrusor contractility, we found that in patients regardless of BOO or non-BOO and DO or non-DO, the BCI decreased over time, with similar trends. Although it is possible the change of BCI might not be obviously seen in early young age, this study provided a scientific evidence by urodynamic study in patients after middle-age, which has not been studied in the literature. This finding suggests that in patients with LUTS due to BOO or DO, with adequate medical treatment, detrusor contractility is not aggravated over 10 or more years of follow-up.

To our knowledge, our report is the first one that focused on detrusor contractility in patients with or without BOO and with or without DO who were follow-up of more than 10 years. Shin *et al*. analyzed age-associated changes in urodynamic parameters and found detrusor contractility decreased with age in women with stress urinary incontinence^9^. Pfisterer *et al*. reported detrusor contractility, bladder sensation, and urethral pressure declined with age in community-based women volunteers^10^.

Aging itself could cause the detrusor contractility to decrease. The evidence from basic and clinical studies have shown the pathophysiology of this issue. In an animal study, age-related alterations in caveolae and caveolin protein expression in elderly mice resulted in a decrease in bladder contractility^11^. This age-related impairment of contractility is usually associated with structural changes in the detrusor muscle (dense bands), decreased axonal content, a reduced detrusor to collagen ratio, and changes in muscarinic receptors^12^. Another study showed genomic changes associated with aging might contribute to the age-related bladder functional deterioration in mice^13^. Additionally, aging is associated with impairment of blood vessel function, which may play an important role in the development of bladder dysfunctions^14^. These factors all could lead the detrusor contractility impairment.

DU is one such age-related change in the urinary bladder. In both sexes, DU increased with age, and it was usually accompanied by DO and BOO^15^. Our previous study showed that women with voiding dysfunction and men with BOO have higher incidence of DU and DO than normal group^16,17^. DU is defined as a contraction of reduced strength or duration, resulting in prolonged bladder emptying or a failure to achieve complete bladder emptying within a normal period^18^. In this study, we did not enroll patients with DU, but we found the PVR increased and voiding efficacy decreased with time. We also found that bladder contractility in patients with BOO and DO decreased over time.

Another interesting issue is how the factors of BOO affecting detrusor contractility. A previous study has shown that in men with LUTS, detrusor contraction power parameters and BCI increase with rising BOO grade^19^. However, men with long-term BOO due to BPH have increased detrusor collagen content, which is associated with decreased bladder compliance, DO, and urinary retention^20^. Severe BOO may lead to detrusor function decompensation. Growing evidence has shown that just as in BOO secondary to BPH, these anatomical and functional changes may induce significant alterations in the morphology and physiology of the urothelium and detrusor muscles^21^. Nonetheless, we found that the change in detrusor contractility was similar in men with moderate BOO treated with an alpha-blocker and a 5α-reductase inhibitor compared to those without BOO if they had regular follow up at the clinic.

In this study, patients with BOO had significantly greater increase in PVR volume than patients without BOO as they aged, suggesting that detrusor contractility decreases with time while urethral resistance is higher in BOO group. However, the BCI in men without BOO also decreased with ageing significantly. About three-quarters of men with symptomatic BOO have LUTS that improve with alpha-blocker monotherapy. Residual storage LUTS after alpha-blocker treatment is usually attributable to bladder dysfunction such as DO or detrusor hyperactivity with impaired contractility, and an antimuscarinic agent should be added to the treatment^22^. Men with BOO who had persistent storage symptoms after initial alpha-blocker monotherapy revealed that bladder dysfunction without BOO was present in one third of the patients^23^. In men with a low Qmax and increased PVR after long-term medical therapy, surgical intervention should be cautiously considered because these patients might not have true BOO but could have impaired detrusor contractility upon aging.

Another interesting finding is that in women with DO on antimuscarinic treatment, the change in detrusor contractility did not differ significantly from patients without DO. In clinical practice, we usually worried about antimuscarinic inhibit bladder contraction which might cause voiding difficulty. It is not surprising to find that FSF, FS, US, CBC, and voided volume decreased significantly in patients with DO over time compared to patients without DO. Nevertheless, the changes in Qmax, voided volume, BCI, and PVR volume did not differ significantly between the DO and non-DO groups. Antimuscarinic agents have been an integral part of the standard treatment of DO and overactive bladder. Although they were designed to reduce the strength of overactive detrusor contractions, in the clinical setting, we found that at therapeutic doses, they do not affect the detrusor contractility^24^. Antimuscarinic agents act on muscarinic receptors on the urothelial cells in the bladder and on structures in the suburothelium (interstitial cells, nerves). Therefore, clinically, antimuscarinic agents act mainly during the storage phase, decreasing urgency sensation, and increasing bladder capacity, reducing voiding difficulties^25^. Researchers also reported that antimuscarinic agents had no significant effect on bladder contractility in patients with idiopathic DO, and the therapeutic effects were due to improvements in sensory variables^26^. This finding is compatible with our study, which showed that patients with DO treated with long-term antimuscarinic therapy did not have decreased detrusor contractility. Our results support the safety of long-term antimuscarinic agent use in patients with DO.

The first limitation of this study is its retrospective nature, and the ratio of men to women is not equal. As we mentioned, this is a retrospective study using valid videourodynamic study for analysis. Therefore, not all patients were included in this study unless they had a baseline and follow-up videourodynamic study data. BOO is much more often seen in men so the sex differences showed a stark contrast, and it might make sexual related results unavoidably. Additionally, patients were excluded during the follow-up period if they underwent any surgical intervention which could have caused selection bias. Secondly, the underlying comorbidity was not discussed, and this could have affected the detrusor contractility. Nevertheless, this longitudinal, long-term study provides evidence that detrusor contractility decreases over time in both men and women. Patients with LUTS due to accurately diagnosed BOO and DO who receive adequate treatment usually have detrusor contractility similar to general population after follow-up of 10 or more years.

In conclusion, detrusor contractility decreases in men and women with age as shown by 10 or more years of follow-up. In men with and without BOO, the change of decreased detrusor contractility was also similar. Women with DO on long-term antimuscarinic treatment have significantly increased bladder sensation but having a similar decrease in detrusor contractility as women without DO.

## Methods

This study retrospective investigated a cohort of male and female patients with LUTS follow-up at clinic for more than 10 years. This study analyzed the changes of detrusor contractility after a 10-year span, in a cohort of patients with VUDS at baseline and >10 years, including 49 male patients without BOO and 54 with BOO, and in 117 female patients with (n = 43) and without (n = 74) urodynamic DO. We have added this statement in the beginning of Methods section.

We obtained approval for the study from the Research Ethics Committee at Tzu-Chi Medical Foundation (IRB101-104). All methods were carried out in accordance with relevant guidelines and regulations. Exclusion criteria included neurogenic lower urinary tract dysfunction (NLUTD), such as cerebrovascular accident, dementia or Alzheimer disease, multiple sclerosis or Parkinson disease, spinal cord injury or malformation resulting in gross neuropathy, detrusor-sphincter dyssynergia. Patients who underwent previous pelvic surgery, lower urinary tract surgeries and bladder treatment during the follow-up period, such as transurethral surgery or bladder instillation therapy were also excluded.

All patients received VUDS at the first time whch was difined as baseline and repeat VUDS at >10 years later to compare the changes of parameters. We recorded the intravesical pressure (Pves), intra-abdominal pressure (Pabd) and calculate the PdetQmax. VUDS findings and the interpretation of the results were documented immediately after the study was finished. Filling cystometry data including bladder FSF, FS, US, and Pdet Qmax of voiding and CBC, compliance data were analyzed. Uroflowmetry recorded Qmax, voided volume. PVR, BCI (BCI = pdetQmax + 5 × Qmax), and BOOI (BOOI = PdetQmax − 2 × Qmax) were calculated. The terminology used in this study is that recommended by the International Continence Society^27,28^. Cystometrography and cystography were concomitantly performed and a C-arm cinefluoroscopy was performed to visualize the bladder neck and urethra during the filling and voiding phases. BOO in women was defined as the radiologic evidence of bladder outlet narrowing plus a PdetQmax greater than 35 cm H2O and a Qmax less than 15 mL/s or a PdetQmax greater than 40 cm H2O^29^. On the other hand, male patients with BOO was defined as a BOOI greater or equal to 40, or a pressure-flow study showing a PdetQmax greater or equal to 50 cmH_2_O. In patients with equivocal pressure flow results, the features of the bladder neck, prostatic urethra, and external sphincter on voiding cystourethrography were used for the diagnosis of BOO^30^.

### Statistical analysis

The urodynamic variables analyzed in this study included the first FSF, FS, US, CBC, bladder compliance, Qmax, PdetQmax, and PVR, BCI and BOOI. Descriptive statistics are expressed as means ± standard deviations (SDs) or percentages. *P* values less than 0.05 were considered significant. All calculations were performed using SPSS for Windows, version 16.0.

### Informed consent

Informed consent was obtained from all individual participants included in the study.
